# TOX Acts as a Tumor Suppressor by Inhibiting mTOR Signaling in Colorectal Cancer

**DOI:** 10.3389/fimmu.2021.647540

**Published:** 2021-04-09

**Authors:** Mengdi Yang, Qianru Huang, Changcan Li, Zhiyuan Jiang, Jing Sun, Zhiyu Wang, Rui Liang, Dan Li, Bin Li, Hui Zhao

**Affiliations:** ^1^Department of Internal Oncology, Shanghai Jiao Tong University Affiliated Sixth People's Hospital, Shanghai, China; ^2^Shanghai Institute of Immunology, Shanghai Jiao Tong University School of Medicine, Shanghai, China; ^3^Department of Immunology and Microbiology, Shanghai Jiao Tong University School of Medicine, Shanghai, China; ^4^Henan Key Laboratory of Digestive Organ Transplantation, Department of Hepatobiliary and Pancreatic Surgery, The First Affiliated Hospital of Zhengzhou University, Henan, China; ^5^Institute of Arthritis Research, Guanghua Integrative Medicine Hospital, Shanghai, China

**Keywords:** TOX, rapamycin, PD1, colorectal cancer, immunotherapy

## Abstract

The treatment and prognosis of advanced colorectal cancer (CRC) remain a challenging clinical research focus. Here, we describe a new CRC tumor suppressor and potential therapeutic target: thymocyte selection associated high mobility group box (TOX) protein. The expression of TOX was lower in CRC than para-CRC. With the increase of tumor stage, TOX expression decreased, indicating the presence of TOX relates to better overall survival (OS). TOX suppressed the mechanistic target of rapamycin kinase (mTOR) signaling to inhibit cell proliferation, migration, invasion, and change the epithelial-mesenchymal transition (EMT) process. In addition, TOX promoted apoptosis. As tumor mutation burden and tumor microenvironment play vital roles in the occurrence and development of tumors, we analyzed the TOX expression in the immune microenvironment of CRC. The high TOX expression was negatively correlated with TumorPurity. Moreover, it was positively related to ImmuneScore, StromalScore, microsatellite instability (MSI) status, and Consensus Molecular Subtypes (CMS) 3 typing. Based on gene set enrichment analysis (GSEA), the reduced expression of TOX activated mTOR. We found rapamycin, a mTOR inhibitor, partly inhibited cell proliferation, invasion, and migration in shTOX HCT116 cells. Lastly, TOX suppressed tumorigenesis and lung metastasis of CRC *in vivo*. Rapamycin alone or combined with PD1 inhibitor is more effective than PD1 inhibitor alone in a tumor model. Taken together, these findings highlight the tumor-suppressive role of TOX in CRC, especially in MSI CRC, and provide valuable information that rapamycin alone or combined with PD1 inhibitor has therapeutic potential in CRC.

## Introduction

Colorectal cancer (CRC) is the third most common type of malignant tumor (1, 2). Surgical treatment of early-stage CRC improves prognosis, but the treatment of patients with mid-advanced CRC remains a challenging clinical research focus (3).

The tumor microenvironment (TME) plays an important role in the occurrence and development of tumors (4, 5). Thus, modulating the TME has therapeutic potential, particularly in regards to immune modulation. Immunotherapy is a favored option for treating advanced CRC (6). Knowing the status of microsatellite DNA is essential for immunotherapeutic treatment decisions because CRC tumors with microsatellite instability (MSI) may have more tumor mutation burden (TMB) and a good response to immunotherapy (7). However, not all MSI patients respond to immunotherapy, possibly due to immunosuppressive cells or exhausted T cells in the immune microenvironment (8). Thus, it is worth investigating which MSI patients respond most effectively to immunotherapy. Tools to identify and treat such patients are urgently needed.

Li et al. found that repurposing of drugs targeting cancer metabolism was a promising strategy to improve immunotherapy *via* metabolic reprogramming of the TME (9). Rapamycin, a specific inhibitor of the mammalian target of rapamycin (mTOR), is already a useful cancer treatment choice (10), possibly *via* its effects on the immune system. The phosphatidylinositol-4,5-bisphosphate 3-kinase catalytic subunit alpha (PI3K)/RAC-alpha serine/threonine kinase 1 (AKT)/mechanistic target of rapamycin kinase (mTOR) signaling pathway increases the production of free fatty acids (11) that are more effectively consumed by regulatory T cells than effector T cells, generating an immunosuppressive TME that underlies resistance to immune checkpoint inhibition (12).

Identifying additional factors that function in T cells may also improve immunotherapy. Thymocyte selection-associated high mobility group box (TOX) is a nuclear DNA binding protein that regulates cell growth, DNA repair, and genomic instability in T cell acute lymphoblastic leukemia (13). Previous studies have shown that TOX is hypermethylated in 43% of breast tumors (14). Kim et al. demonstrated that VEGF-A drove TOX-dependent T cell exhaustion in anti-programmed cell death 1 (PD1) resistant microsatellite stable (MSS) tumors (15). However, no published studies have yet described the function and mechanism of TOX in CRC cells.

In this study, the clinical significance and related mechanisms of TOX in CRC were investigated. Given that TOX inhibits mTOR signaling pathway activation, we also describe the combined effects of rapamycin and PD1 treatment, indicating that rapamycin can be repurposed to improve the immunotherapeutic outcomes of CRC.

## Results

### TOX Is Downregulated in Human CRC Tissues From Patients With Poor Survival

**Figure 1:**
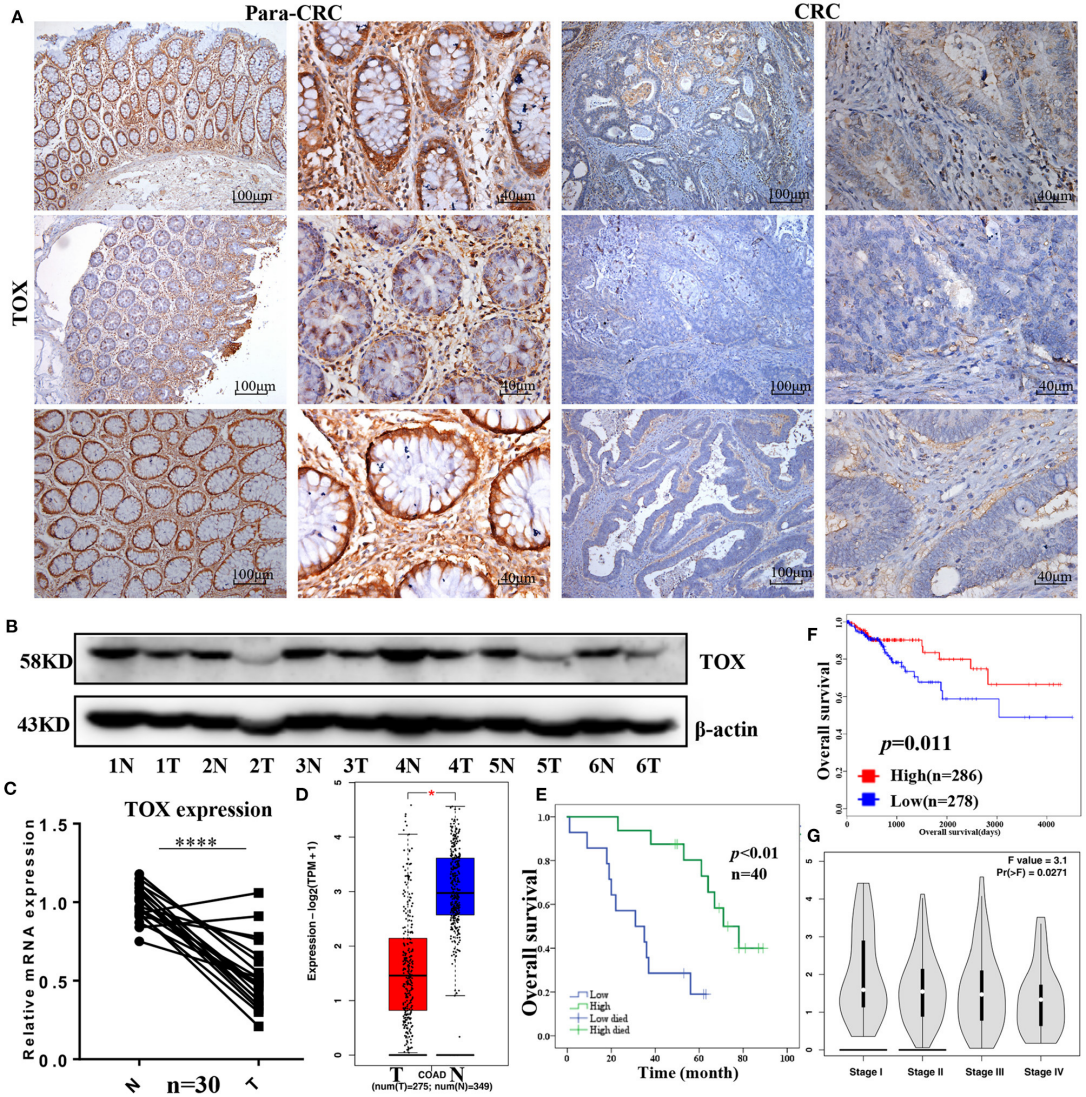
TOX is downregulated in human CRC tissues from patients with poor survival. TOX expression was confirmed in para-CRC and CRC by IHC **(A)**, western blot **(B)**, and qRT-PCR **(C)**. **(D)** Public databases (http://gepia.cancer-pku.cn/) show the TOX expression in tumor (*n* = 275) and normal (*n* = 349) tissues. **(E)** The relationship between TOX expression and CRC patient survival in our study population as well as **(F)** public databases (http://tcoa.cpu.edu.cn/). **(G)** TOX expression was shown in different CRC stages. *p < 0.05, ****p < 0.0001.

Most articles have reported the role of TOX in CD8^+^ T cells (8, 16, 17), but few have tested the function of TOX in tumor cells, especially in CRC. Thus, we first examined the expression of TOX in human CRC tissues. Immunohistochemical semi-quantitative analysis showed that primary CRC tissues (2.15 ± 1.688) had significantly lower levels of TOX protein staining (*p* = 0.004) than adjacent non-tumor tissues (4.45 ± 2.050) (Figure 1A, Table 1). Likewise, western blots indicated TOX protein levels were significantly downregulated in CRC tissues relative to matched para-CRC tissues (Figure 1B). Quantitative real-time PCR (qRT-PCR) comparing 30 CRC and paired para-CRC tissues also presented significantly reduced TOX mRNA level in tumor samples (*p* < 0.0001) (Figure 1C). To further assess TOX expression in CRC, we referred to the public gene expression datasets (http://gepia.cancer-pku.cn/). It is found that the expression of TOX was lower in CRC tumors (*n* = 275) than in non-tumor (*n* = 349) tissues (Figure 1D). These results demonstrate the TOX expression is decreased at the transcriptional and translational levels in CRC samples compared with non-tumor tissues.

**Table 1:** The expression of TOX in CRC and para-CRC tissues was analyzed by chi-square test.

**Tissue sample**	***n***	**Mean ± SD**	**TOX expression**		***p*-value**
			**Low (%)**	**High (%)**	
CRC	40	2.15 ± 1.688	27 (65.9)	13 (33.3)	0.004^*^
Para-CRC	40	4.45 ± 2.050	14 (34.1)	26 (66.7)	

* *p < 0.05 indicates a significant difference*.

### Association Between TOX Expression and Clinicopathological Parameters for CRC

Kaplan–Meier curves were used to assess the association between TOX expression and survival of CRC patients. Patients with low TOX expression in tumors had significantly poorer overall survival (OS) than patients with higher TOX expression in our study population (Figure 1E) as well as in public datasets (http://tcoa.cpu.edu.cn/) (Figure 1F). And TOX expression decreased with increasing tumor grade (http://gepia2.cancer-pku.cn) (Figure 1G). Further, TOX expression was significantly associated with several clinicopathological factors, including age, N stage, American Joint Committee on Cancer (AJCC) stage, vimentin, and p-mTOR expression (Table 2). A Cox proportional hazards model was used for univariate and multivariate analyses of OS. In the univariate analysis, N stage, AJCC stage, TOX, and p-mTOR expression were significantly associated with OS (Table 3). In the multivariate analysis, N stage, AJCC stage, TOX, and p-mTOR expression were independent prognostic factors for OS (Table 4). Thus, TOX expression can serve as a critical predictor for OS of CRC patients.

**Table 2:** Correlation between TOX expression and clinicopathological features were analyzed by chi-square test, adjusted chi-square test, or Fisher's exact test.

**Clinicopathological features**	**Total (*n* = 40)**	**TOX (*****n*** **= 40)**		***P*-value**
		**Low (*n* = 27) (%)**	**High (*n* = 13) (%)**	
**Age, years**				
<65	15 (37.5)	7 (25.9)	8 (61.5)	0.041^*^
≥65	25 (62.5)	20 (74.1)	5 (38.5)	
**Gender**				
Male	17 (42.5)	10 (37.0)	7 (53.8)	0.314
Female	23 (57.5)	17 (63.0)	6 (46.2)	
**T stage**				
T1+T2	17 (42.5)	12 (44.4)	5 (38.5)	0.72
T3+T4	23 (57.5)	15 (55.6)	8 (61.5)	
**N stage**				
N0	19 (47.5)	9 (33.3)	10 (76.9)	0.017^*^
N1	21 (52.5)	18 (66.7)	3 (23.1)	
**M stage**				
M0	33 (82.5)	20 (74.1)	13 (100.0)	0.07
M1	7 (17.5)	7 (25.9)	0 (0)	
**AJCC stage**				
I+II	23 (57.5)	11 (40.7)	12 (92.3)	0.002^*^
III+IV	17 (42.5)	16 (59.3)	1 (7.7)	
**Location**				
Left colonic	8 (20.0)	5 (18.5)	3 (23.1)	0.938
Right colonic	26 (65.0)	18 (66.7)	8 (61.5)	0.939
Rectum	6 (15.0)	4 (14.8)	2 (15.4)	0.843
**E-cadherin**				
High	20 (50.0)	16 (59.3)	4 (30.8)	0.176
Low	20 (50.0)	11 (40.7)	9 (69.2)	
**Vimentin**				
High	17 (42.5)	8 (29.6)	9 (69.2)	0.038^*^
Low	23 (57.5)	19 (70.4)	4 (30.8)	
**p-mTOR**				
Positive	17 (42.5)	6 (22.2)	11 (84.6)	0.000367^*^
Negative	23 (57.5)	21 (77.8)	2 (15.4)	

* *p < 0.05 indicates a significant difference*.

**Table 3:** Univariate analysis for overall survival (OS).

**Variable**	**OS**		
	**OR**	**95% CI**	***p*-value**
**Age, years**			
<65	2.914	0.614–13.829	0.178
≥65			
**Gender**			
Male	0.740	0.174–3.136	0.682
Female			
**T stage**			
T1+T2	0.603	0.126–2.946	0.538
T3+T4			
**N stage**			
N0	0.053	0.006–0.471	0.008^*^
N1			
**M stage**			
M0	642858.900	0.000–1.359	0.894
M1			
**AJCC stage**			
I+II	11.015	1.207–100.498	0.033^*^
III+IV			
**Location**			
Left colonic	1.676	0.615–4.566	0.313
Right colonic			
Rectum			
**TOX**			
High	0.032	0.001–0.863	0.041^*^
Low			
**E-cadherin**			
High	0.494	0.098–2.500	0.394
Low			
**Vimentin**			
High	1.566	0.364–6.742	0.547
Low			
**p-mTOR**			
Positive	25.229	1.491–462.915	0.025^*^
Negative			

* *p < 0.05 indicates a significant difference*.

**Table 4:** Multivariate analysis for OS.

**Variable**	**OS**		
	**OR**	**95% CI**	***p*-value**
N stage	0.195	0.065–0.586	0.004
TOX	0.118	0.016–0.875	0.036
AJCC stage	8.358	2.495–27.998	0.001
p-mTOR	33.027	3.183–342.659	0.003

### TOX Expression Is Higher in MSI CRC Than in MSS CRC, Positively Correlates With ImmuneScore and StromalScore, and Negatively Correlates With TumorPurity

**Figure 2:**
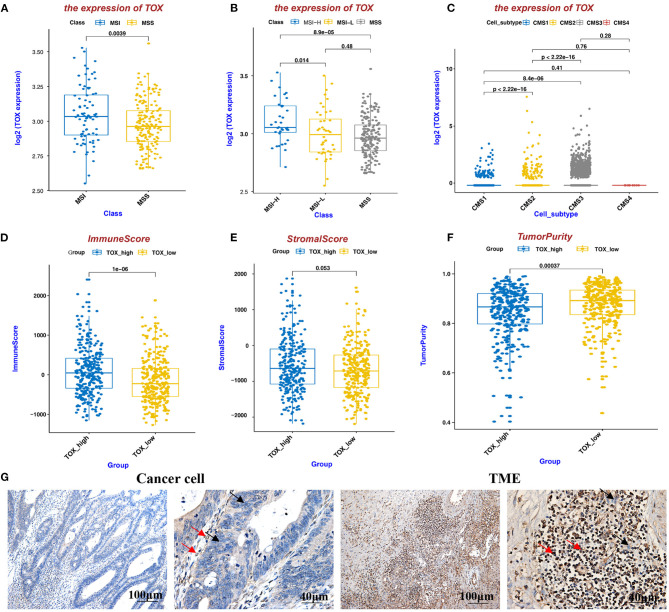
TOX expression is higher in MSI CRC than in MSS CRC, positively correlates with ImmuneScore and StromalScore, and negatively correlates with TumorPurity. **(A,B)** MSI status of TOX-low and TOX-high CRC tumors. **(C)** TOX expression in different CMS subtypes. **(D–F)** The relationship between TOX expression and ImmuneScore **(D)**, StromalScore **(E)**, and TumorPurity **(F)** in CRC patient (inferred by ESTIMATE algorithm). **(G)** TOX expression was shown in cancer cells and the TME by IHC. Black arrows represent tumor cells, red arrows represent immune cells.

Because MSI status acts as a significant role in the choice of CRC treatment, we analyzed MSI status based on TOX expression in data from the Cancer Genome Atlas (TCGA). Patients with high TOX expression were mostly MSI (MSI-H *n* = 36, MSI-L *n* = 44), while those with low expression were mostly MSS (*n* = 179) (*p* < 0.05) (Figures 2A,B). Additionally, we classified TOX expression according to Consensus Molecular Subtypes (CMS) (18). Seventeen thousand four hundred and sixty-nine epithelial cells of tumor tissue were analyzed in the single-cell data set, including 1,201 CMS1 cells, 10,771 CMS2 cells, 5,486 CMS3 cells, and 11 CMS4 cells (19). Most tumors expressing high TOX were type CMS3 (31.40%), which has a good prognosis (Figure 2C). ImmunoScore provides a reliable estimate of the risk of recurrence in patients with colon cancer. Patients with a high ImmunoScore had the lowest risk of recurrence at 5 years (20). TOX expression had a significantly positive association with ImmuneScore (*p* = 1e-06) (Figure 2D). It was also positively correlated with StromalScore (*p* = 0.053) (Figure 2E) and significantly negatively correlated with TumorPurity (*p* = 0.00037) (Figure 2F). Consistently, IHC showed that TOX expression was lower in cancer cells but highly expressed in the TME (Figure 2G). These results suggest that high TOX expression may be essential for immunotherapy.

### TOX Expression Suppresses Cancer Cell Proliferation, Migration, Invasions, and Promotes Apoptosis *in vitro*

**Figure 3:**
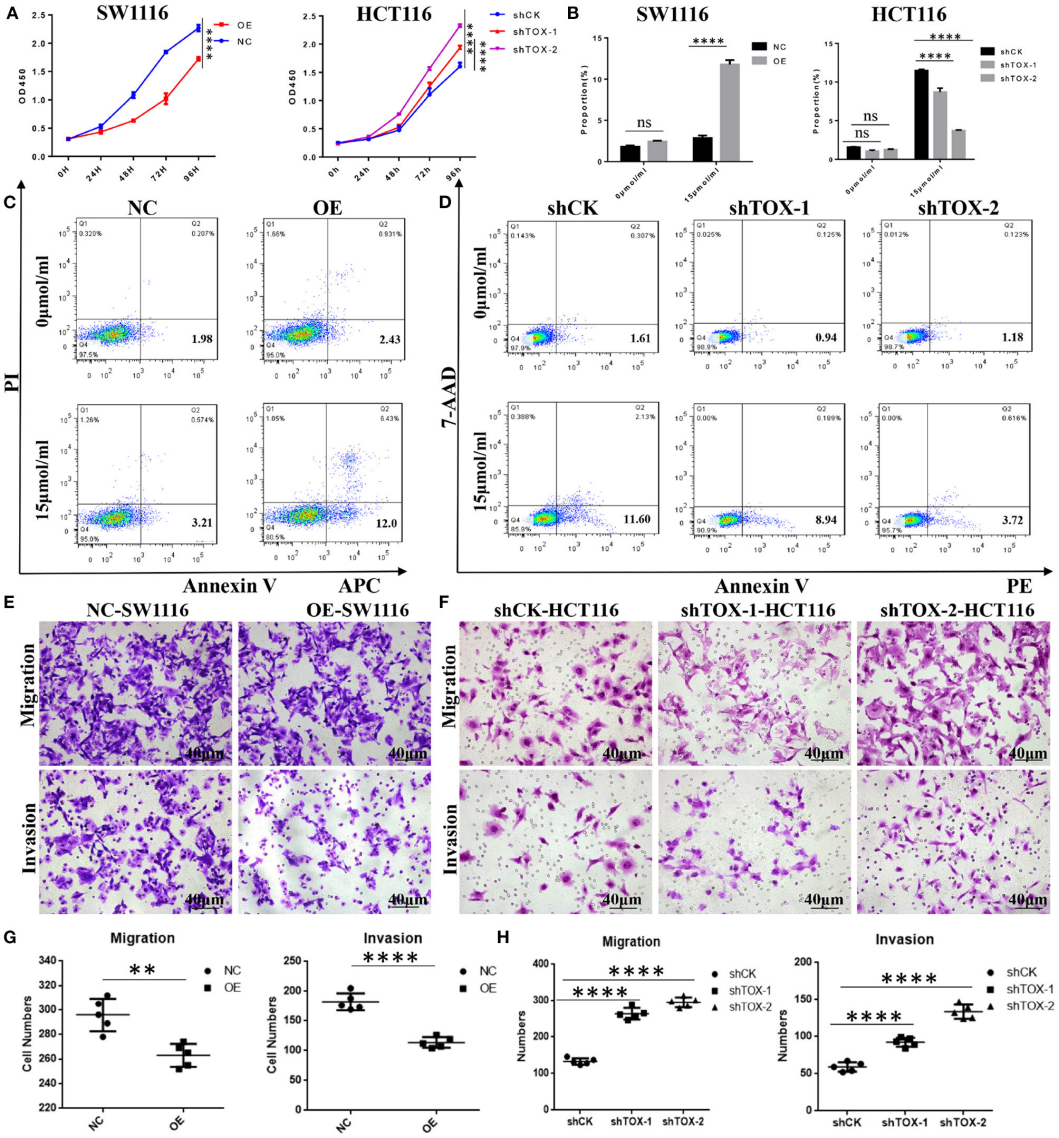
TOX expression suppresses cancer cell proliferation, migration, invasion, and promotes apoptosis *in vitro*. **(A)** Cell proliferation was detected by CCK8 in SW1116 (MSS) and HCT116 (MSI) cells. **(B)** The statistical results of the apoptosis experiment. **(C)** FACS analysis of apoptosis assay in PLVX-Flag and PLVX-Flag-TOX SW1116 cells. **(D)** FACS analysis of apoptosis assay in shCK HCT116 and shTOX HCT116 cells. **(E,G)** Transwell assays show migration and invasion of PLVX-Flag and PLVX-Flag-TOX SW1116 cells. **(F,H)** Transwell assays show migration and invasion in shCK and shTOX HCT116 cells. Each experiment was repeated three times. **p < 0.01, ****p < 0.0001.

To test the effects of TOX on MSI and MSS CRC cell functions, we overexpressed TOX in SW1116 (MSS) cells which are low expressed of TOX and knocked-down TOX in HCT116 (MSI) cells which are highly expressed of TOX (Supplementary Figure 1). Viability assays showed that TOX overexpression significantly inhibited cell expansion and TOX knockdown promoted cell expansion (Figure 3A). We further explored how the presence or absence of TOX expression affects apoptosis induced by cisplatin, a commonly used inducer of apoptotic cell death (21). Overexpression of TOX significantly promoted apoptosis, and knockdown of TOX inhibited apoptosis (Figures 3B–D). Besides, transwell assays were used to assess whether TOX affects cell migration and invasion. Overexpression of TOX significantly suppressed SW1116 cell migration and invasion (Figures 3E,G), whereas knockdown of TOX promoted HCT116 cell migration and invasion (Figures 3F,H). These data show that TOX inhibits cell proliferation, migration, invasion and promotes cell apoptosis in CRC cells.

### TOX Represses the Epithelial-Mesenchymal Transition

**Figure 4:**
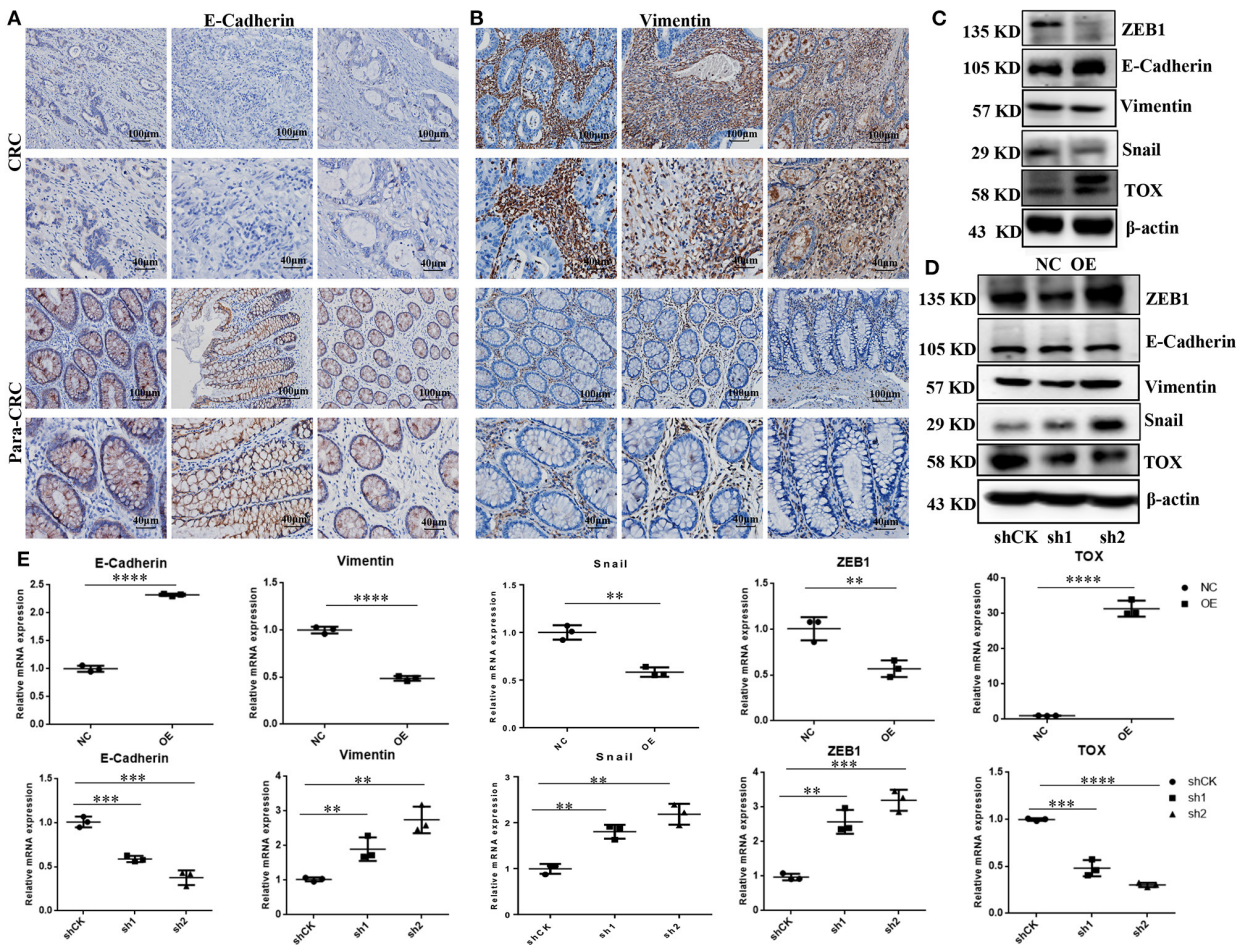
TOX represses the EMT process. IHC shows assay E-cadherin **(A)** and vimentin **(B)** expression in CRC and para-CRC tissues (*n* = 40). **(C)** Western blot shows EMT-related proteins ZEB1, E-cadherin, vimentin, and Snail expression in PLVX-Flag-TOX and PLVX-Flag SW1116 cells. **(D)** Western blot assay shows ZEB1, E-cadherin, vimentin, and Snail in shCK HCT116, shTOX-1 (sh1), and shTOX-2 (sh2) HCT116 cells. **(E)** qRT-PCR shows ZEB1, E-cadherin, vimentin, and Snail expression in PLVX-Flag-TOX, PLVX-Flag SW1116, shCK HCT116, shTOX-1 (sh1), and shTOX-2 (sh2) HCT116 cells. Each experiment was repeated three times. *p < 0.01, ***p < 0.001, ****p < 0.0001.

To explore the molecular mechanisms underlying TOX-mediated attenuation of CRC migration and invasion, we explored the expression of EMT markers in CRC tissues. IHC showed that E-cadherin was lower in CRC tissue than para-CRC tissue (Figure 4A), while vimentin was higher in CRC compared with para-CRC tissue (Figure 4B). Western blots showed E-cadherin expression increased, while Zinc Finger E-Box Binding Homeobox 1 (ZEB1), Snail Family Transcriptional Repressor 1 (Snail) dramatically decreased, and vimentin slightly decreased when TOX was overexpressed in PLVX-Flag-TOX SW1116 cells compared with control PLVX-Flag SW1116 cells (Figure 4C). ZEB1, vimentin, and Snail expression increased, when TOX was downregulated in shTOX HCT116 cells compared with control shCK HCT116 cells (Figure 4D). And qRT-PCR showed E-cadherin expression increased, while ZEB1, vimentin, and Snail dramatically decreased when TOX was overexpressed in PLVX-Flag-TOX SW1116 cells compared with control PLVX-Flag SW1116 cells (Figure 4E). Conversely, E-cadherin expression decreased, while ZEB1, vimentin, and Snail increased, when TOX was downregulated in shTOX HCT116 cells compared with control shCK HCT116 cells (Figure 4E). These results suggest that restoring TOX may be able to reverse EMT in CRC.

### Rapamycin Partly Reverses the Aggressive Phenotype of TOX-Deficient Cells *in vitro*

**Figure 5:**
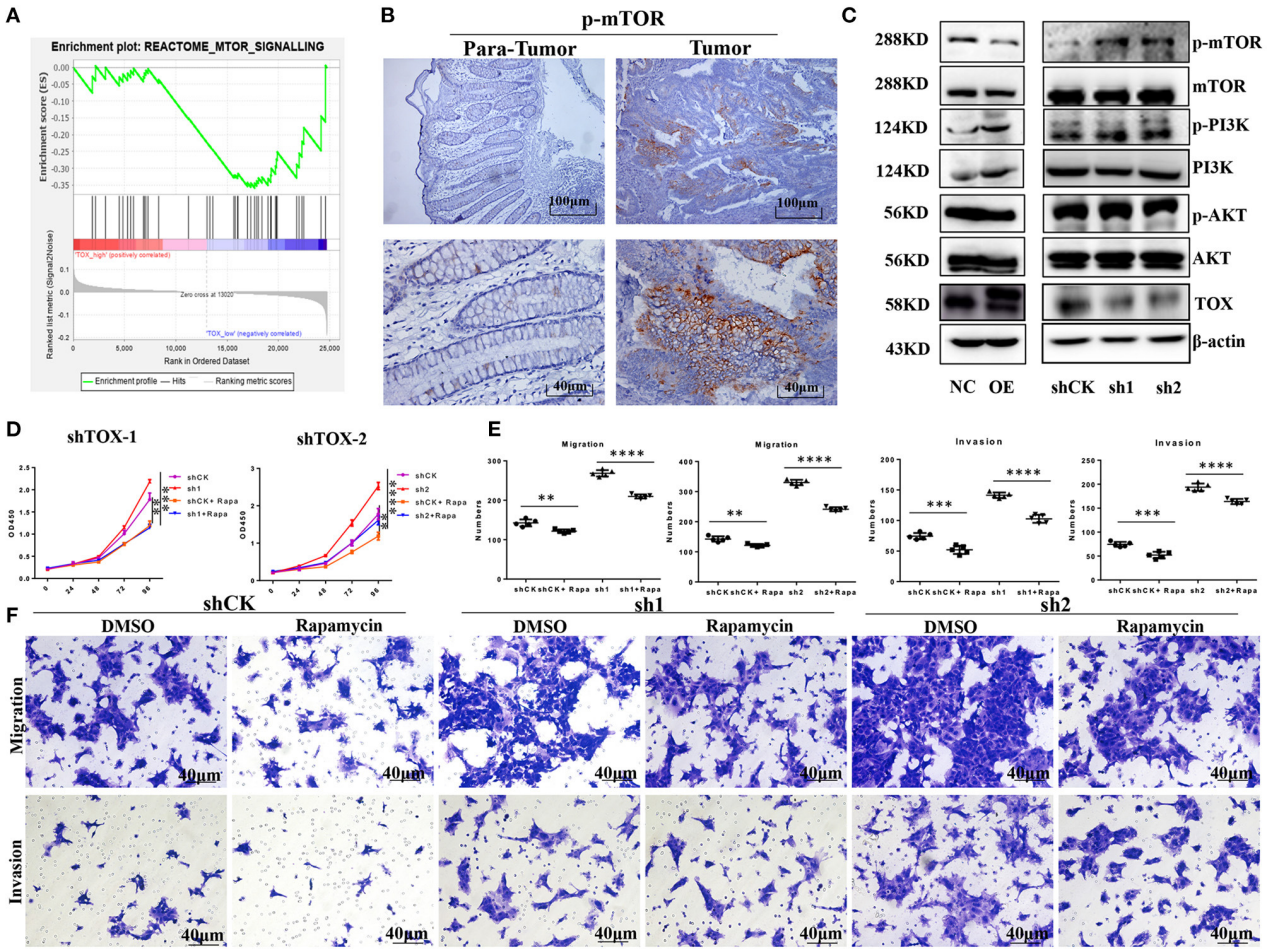
Rapamycin partly rescues the aggressive phenotype of TOX-deficient cells. **(A)** GSEA was used to analyze the mTOR signaling pathway in CRC patients (*n* = 588). **(B)** IHC shows the expression of p-mTOR in CRC (*n* = 40). **(C)** Western blots showed the expression of p-PI3K, PI3K, p-AKT, AKT, mTOR, and p-mTOR in PLVX-Flag-TOX SW1116, PLVX-Flag SW1116 cells, shCK HCT116, shTOX-1 (sh1), and shTOX-2 (sh2) HCT116 cells. **(D)** Cell proliferation was measured in shCK HCT116 and shTOX HCT116 cells with or without rapamycin treatment by CCK8. **(E,F)** Cell migration and invasion were measured with or without rapamycin in shCK HCT116, shTOX-1 (sh1), and shTOX-2 (sh2) HCT116 cells by Transwell. Each experiment was repeated three times. **p < 0.01, ***p < 0.001, ****p < 0.0001.

To explore the mechanism by which TOX inhibits tumor cell proliferation, migration, invasion, and EMT, we performed gene set enrichment analysis (GSEA) on TCGA data (*n* = 588). Proteins involved in mTOR signaling were enriched in CRC cells with low TOX expression (Figure 5A). Previous studies have shown that mTOR regulates EMT, motility, and metastasis of CRC (22). Using IHC, we found that CRC tumors had higher levels of mTOR activation (p-mTOR) than adjacent non-tumor tissues (Figure 5B). Western blots showed that overexpressing or downregulating TOX did not change phosphorylation of PI3K or AKT modifications in PLVX-Flag-TOX SW1116 cells or shTOX HCT116 cell lines compared with corresponding controls (Figure 5C). However, p-mTOR was significantly downregulated in PLVX-Flag-TOX SW1116 cells and upregulated in shTOX HCT116 cells compared with the corresponding controls. To check the function of mTOR in CRC, we inhibited mTOR expression in shTOX HCT116 cells with rapamycin, a highly efficient mTOR pathway inhibitor. Rapamycin partly reversed the TOX-loss induced cell expansion (Figure 5D), migration, and invasion (Figures 5E,F). Our results suggest a significant negative correlation between the expression of TOX and mTOR activation levels in CRC tissues, indicating rapamycin may be applied to treat CRC with low TOX expression.

### TOX Acts as a Tumor Suppressor by Inhibiting Tumorigenesis and Metastasis *in vivo*

**Figure 6:**
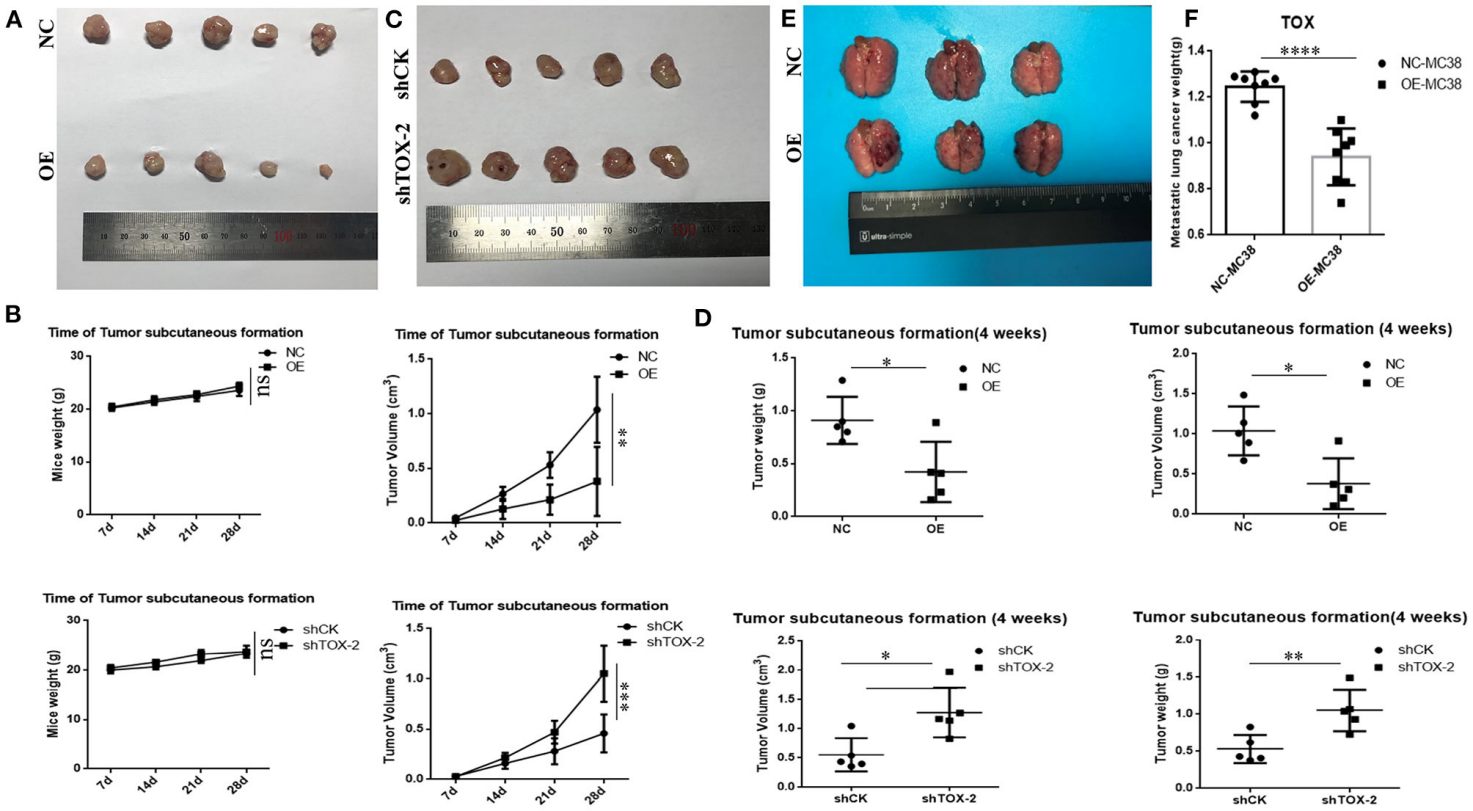
TOX acts as a tumor suppressor by inhibiting tumorigenesis and metastasis *in vivo*. **(A)** Tumor samples of PLVX-Flag SW1116 (negative control, NC) and PLVX-Flag-TOX SW1116 (overexpression, OE) cells were shown (4 weeks). **(B)** Bodyweight and tumor volume of NC and OE group were shown. **(C)** Tumor samples of GFP-shCK HCT116 and GFP-shTOX-2 HCT116 cells were shown (4 weeks). **(D)** Bodyweight and tumor volume of GFP-shCK HCT116 and GFP-shTOX-2 HCT116 group were shown **(E)** Lung metastasis sites of injecting with PLVX-Flag MC38 and PLVX-Flag-*Tox* MC38 cells were shown (3 weeks). **(F)** Lung weights of injecting with PLVX-Flag MC38 or PLVX-Flag-*Tox* MC38 cells were shown (3 weeks). *p < 0.05, **p < 0.01, ****p < 0.0001, ns: not significant.

When separately injected PLVX-Flag SW1116 cells and PLVX-Flag-TOX SW1116 cells into nude mice, PLVX-Flag SW1116 cells produced larger subcutaneous tumors, both by weight and volume, than PLVX-Flag-TOX SW1116 cells (*p* < 0.05; Figure 6A). However, it does not affect the weight of mice (Figure 6B). Conversely, both tumor weight and tumor volume were greater for GFP-shTOX-2 HCT116-injected mice than control GFP-shCK HCT116-injected mice (*p* < 0.05; Figure 6C), without affecting mouse body weight (Figure 6D). Because TOX may inhibit EMT and TOX expression decreases with increasing AJCC stage, we also delivered engineered cancer cells by tail vein injection to test the influence of TOX on tumor metastasis. Flag-MC38-injected C57BL/6 mice (*n* = 8) had more metastasis sites (Figure 6E, Supplementary Figure 2) and greater lung mass (Figure 6F) than Flag-*Tox* MC38-injected C57BL/6 mice (*n* = 8). These findings indicate that TOX inhibits tumor formation and metastasis.

### Rapamycin or PD1 Inhibition Suppresses Tumorigenesis

**Figure 7:**
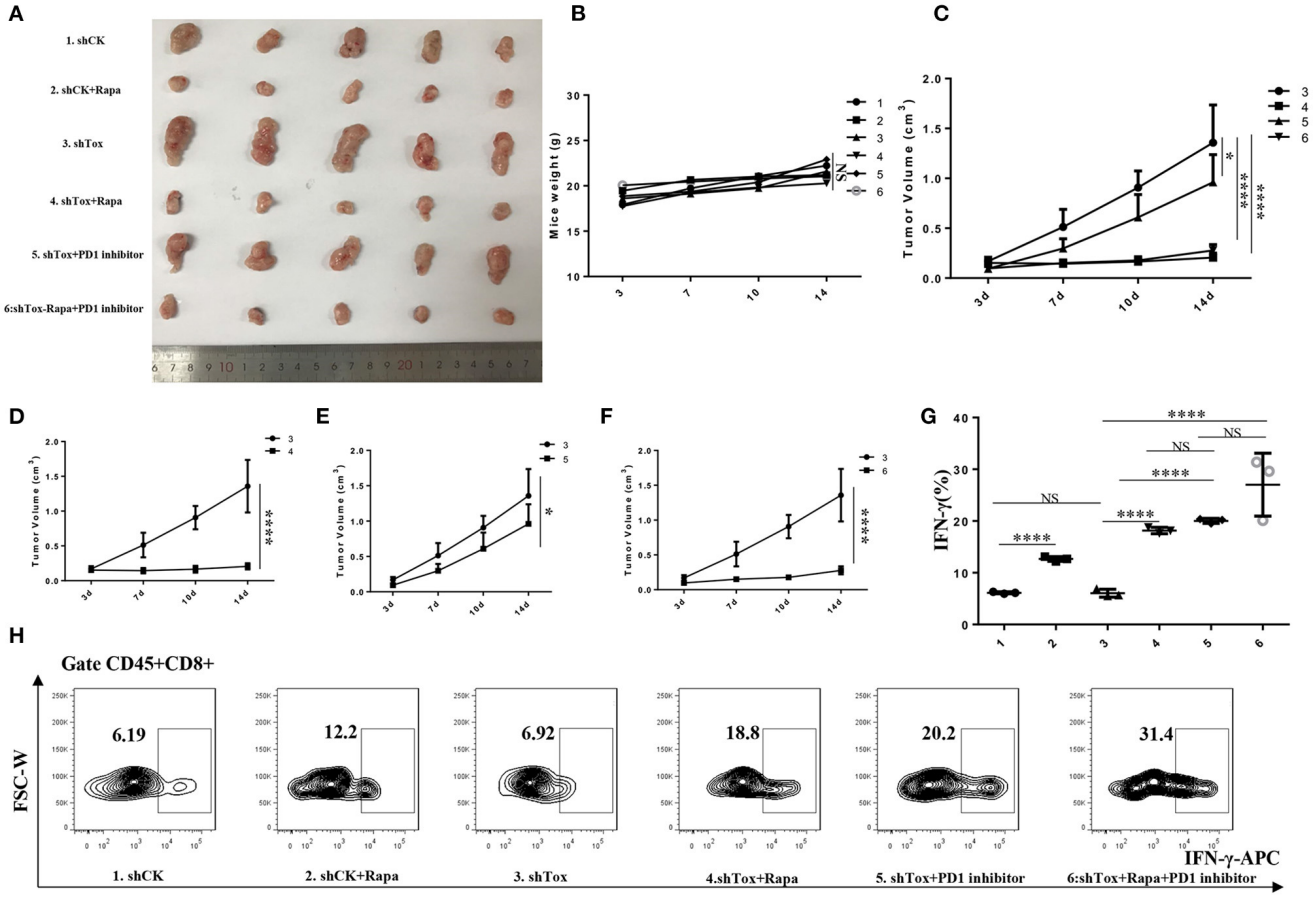
Rapamycin or combined with PD1 inhibition suppresses tumorigenesis. **(A)**. Tumor samples of 6 groups were shown (1. shCK, 2. shCK+rapamycin, 3. *shTox* MC38, 4. *shTox*+rapamycin, 5. *shTox*+PD1 inhibitor, 6. *shTox*+rapamycin+PD1 inhibitor). **(B)**. Mice weights of 6 groups were shown. **(C)**. The tumor volumes of group 3–6 were shown. The tumor volumes of mice group 3–4 **(D)**, 3–5 **(E)** and 3–6 **(F)** were shown **(G,H)**. The secretion of IFN-γ by CD8^+^ T cells in 6 groups mice were shown by FACS. *p < 0.05, ****p < 0.0001, NS: not significant.

To test the therapeutic effect of rapamycin on mice with low *Tox* expression, we injected shCK or *shTox* MC38 cells subcutaneously into C57BL/6 mice, and tumors formed 3 days later; we then injected mice, respectively, with rapamycin, PD1 inhibitor, and rapamycin combined PD1 inhibitor to treat the tumors for 2 weeks (Figure 7A). Rapamycin (Figure 7D), PD1 inhibitor (Figure 7E), and rapamycin combined with PD1 inhibitor (Figure 7F) treatment significantly reduced *shTox* MC38 group tumor size compared with the shCK MC38 group (Figure 7C). The combined treatment cannot significantly reduce tumor volume compared with rapamycin, but significantly reduced tumor size in PD1 inhibitor treatment alone *shTox* MC38-injected groups (Figure 7C). However, there is no statistical difference in mice body weight between these six groups (Figure 7B). To further verify the effects of rapamycin and PD1 inhibition *in vivo*, we conducted an immune microenvironment analysis on each group of mice tissues. We found rapamycin and PD1 inhibitor can significantly increase the secretion of IFN-γ in CD8^+^ cells (Figures 7G,H). Collectively, these results confirm that rapamycin or PD1 inhibition suppress tumorigenesis, likely in part by regulating signaling downstream of TOX and IFN-γ secretion from CD8^+^ T cells.

## Materials and Methods

### Patients

The studies involving human participants were reviewed and approved by the Ethics Committee of Shanghai Jiao Tong University Affiliated Sixth People's Hospital. All patients provided informed consent for their participation. From May 2013 to August 2020, 40 patients with CRC who underwent tumor resection surgery at Shanghai Jiao Tong University Affiliated Sixth People's Hospital (Shanghai, China) were enrolled in this study. CRC classification was based on the 2015 version National Comprehensive Cancer Network guidelines (23). All patients were followed for more than 5 years. Patient inclusion criteria were: no other primary cancer, the pathological diagnosis of CRC, availability of samples for subsequent analyses, and willingness for follow-up.

### Immunohistochemical Assay (IHC)

Paraffin sections were subjected to antigen retrieval for 20 min, and incubated with antibodies against TOX (#ab155768, 1:500; Abcam, USA), p-mTOR (#5536, 1:100; CST, USA), vimentin (#5741, 1:200; CST, USA), and E-cadherin (#3195, 1:400; CST, USA) separately. Then the paraffin section was washed, and incubated with biotinylated secondary antibodies (Kirkegaard & Perry Laboratories Inc., Gaithersburg, MD, USA). Fromowitz's criterion was used for semi-quantitative assessment (24). We did a positive control and a negative control which used corresponding isotype antibody. Both the staining intensity and the staining degree were assessed in a semi quantitative analysis. Intensity of staining was graded as follows: negative = 0, weak positive = 1, moderate positive = 2, and strong positive = 3. The following system was employed to score the percentage of positive tumor cells: 0–5% = 0, 5–25% = 1, 26–50% = 2, 51–75% = 3, >75% = 4. Thus, the minimum score is 0, and the maximum score is 7. We define three points as cutoff point. 0–3 points as low expression group, 4–7 points as high expression group. Two pathologists who were blinded to patient information analyzed the protein staining.

### Quantitative Real-Time Polymerase Chain Reaction

RNA from tissues and cells was extracted using TRIzol reagent (Sigma-Aldrich, St. Louis, MO, USA). QRT-PCR was performed using the PrimeScript RT Reagent Kit (TaKaRa Bio, Shiga, Japan) for reverse transcription and SYBR Premix Ex Taq (TaKaRa Bio). 7900HT Fast Real-Time PCR System (Applied Biosystems, Foster City, CA, USA) was used for qRT-PCR. The conditions included 40 PCR cycles (95°C for 5 s and 60°C for 30 s) after initial denaturation (95°C for 5 min). Gene expression was normalized to β*-actin*. PCR primers are listed in Table 5. All experiments were replicated three times.

**Table 5:** Primers used in the study.

**Genes**	**Sequence**
TOX-forward	GTGATGCCAGATATACGAAACCC
TOX-reverse	AGCTGTGACTGGTTAATGGTAGT
E-cadherin-forward	CGAGAGCTACACGTTCACGG
E-cadherin-reverse	GGGTGTCGAGGGAAAAATAGG
Snail-forward	ACTGCAACAAGGAATACCTCAG
Snail-reverse	GCACTGGTACTTCTTGACATCTG
Vimentin-forward	GACGCCATCAACACCGAGTT
Vimentin-reverse	CTTTGTCGTTGGTTAGCTGGT
ZEB1-forward	CAGCTTGATACCTGTGAATGGG
ZEB1-reverse	TATCTGTGGTCGTGTGGGACT
β-actin-forward	GGACTTCGAGCAAGAGATGG
β-actin-reverse	GCACTGTGTTGGCGTACAG
*shTox*-1	CCCTGAAATCACAGTCTCCAA
*shTox*-2	CGATGATACCTCTAAGATCAA
*shTox*	GTCAACTCAAAGCCGTCAGTA
TOX clone-forward	ATGGACGTAAGATTTTATCCACCTC
TOX clone-reverse	CAAGTAAGGTACAGTGCTTTGTCC
*Tox* clone-forward	ATGGACGTAAGATTTTATCCTCCTC
*Tox* clone-reverse	TCAGGTGAGATACAGCGCTTTGT

### Western Blot

CRC and para-CRC tissue samples or cancer cell lines were lysed in RIPA buffer and resolved by SDS-PAGE. Resolved proteins were transferred to polyvinylidene fluoride membranes and incubated with antibodies against TOX (#ab155768, 1:1,000; Abcam, USA), EMT markers (E-cadherin #3195, Snail #3879, and vimentin #5741, 1:1,000, CST, USA; ZEB1 #21544-1-AP, 1:1000 Proteintech, China), and PI3K/AKT/mTOR pathway molecules (p-PI3K #4228, PI3K #4249, p-AKT, AKT, mTOR #2983, p-mTOR #2974, 1:1,000; CST, USA). Membranes were incubated with horseradish peroxidase-conjugated secondary antibodies (Jackson ImmunoResearch, West Grove, PA, USA). Proteins were visualized with ECL Plus reagent (Millipore, Jaffrey, NH, USA) and normalized to β-actin (Proteintech, China, #66009-1, 1:1000). All experiments were replicated three times.

### Bioinformatics Analysis

An online tool was used to graph survival curves (http://tcoa.cpu.edu.cn/). CRC clinical and expression data (sample numbers: COAD, *n* = 430 and READ, *n* = 158) were downloaded from TCGA datasets (https://www.cancer.gov/tcga). The “DESeq” (1.24.0) “survival” (2.44.1.1), and “estimate” (1.0.13) packages were used for analysis in R. Comparisons between groups were performed using Wilcoxon test. Besides, a single-cell data GSE132465 was analyzed by Seurat (19). We define median TOX expression as cut-off point (median: 7.8592; high group: *n* = 294; low group: *n* = 294). ImmuneScore, StromalScore, and TumorPurity were inferred by the ESTIMATE algorithm (25).

### Cell Culture

CRC cell lines (SW1116, HCT116, MC38) were purchased from the Cell Bank of Type Culture Collection of the Chinese Academy of Sciences (Shanghai, China). DMEM supplemented with 1% penicillin/streptomycin and 10% FBS were used to culture these CRC cell lines with 5% CO_2_ at 37°C. To inhibit mTOR, cells at 80% confluence were treated with rapamycin (Selleck, S1039, 5 μmol/mL) for the indicated times.

### Lentivirus Packaging

HEK293T cells at 70–80% confluence were washed with PBS, and fresh OPTI-MEM (5.4 mL) was added in 10cm dishes. Lentiviral packaging mixtures were prepared by mixing 600 μL OPTI-MEM, 72 μL of polyethyleneimine (PEI), and 24 μg of the relevant plasmids, including 12 μg of the target plasmids, 10.68 μg of dR8.9, and 1.32 μg of VSV-G. The mixture was allowed to stand for 10 min before addition to cells. The medium was changed 4–6 h post-transfection, and the supernatant was collected at 48 and 72 h post-transfection.

### Cell Migration and Invasion Assays

CRC cells that cultured in FBS-free media overnight were seeded into the upper chambers of uncoated (to assess cell migration) or Matrigel-coated (to assess invasion) transwell (Corning Inc., Corning, NY, USA). Cells were incubated for 24–48 h, fixed in methanol, and stained with crystal violet (Beyotime, Beijing, China). Cells in five random fields from the bottom of the membranes were counted. All experiments were replicated three times.

### Cell Proliferation Assay

The CCK-8 assay (Dojindo, Kumamoto, Japan) was used to evaluate cell proliferation. CRC cells were cultured in 96-well plates for 0, 24, 48, 72, and 96 h. At each time point, 100 μL of CCK-8 was added and incubated for 2 h at 37°C. Absorbance was measured at 450 nm by a microplate reader (BioRad, Hercules, CA, USA). Experiments were replicated three times.

### Flow Cytometry

For apoptosis assay, CRC cells were harvested, and apoptosis was assessed by flow cytometry using an Annexin V Apoptosis Detection Kit (eBioscience) at 48 h post-transfection. For TME immune microenvironment analysis, subcutaneous tumors were cut into 2-mm^3^ pieces. The tissues were digested and incubated with collagenase D for 30 min. All cells were washed in PBS with 2% FBS. Then the cells were stimulated with PMA (50 ng/mL), ionomycin (1 mM), Golgi Stop, and Golgi Plug for 4 h before cytokine detection. Next, cells were incubated with Viability Dye eFluor 780 (eBioscience, 1:1,000, #65-0865-14) and antibodies TCR β chain (Biolengend, 1:200, #109221), CD8 (BD, 1:200, #563898), CD45 (eBioscience, 1:200, #11-0451-85), IFN-γ (eBioscience, 1:200, #12-7311-82). All samples were run on the BD LSRFortessa Flow Cytometer (BD Biosciences), and FlowJo software (TreeStar, Ashland, OR, USA) was used to analyze the data.

### Animal Experiments

The animal study was reviewed and approved by the Animal Ethics Committee of Shanghai Jiao Tong University affiliated Sixth People's Hospital. Mice were purchased from the Shanghai SLAC Laboratory and were bred under specific pathogen-free conditions. PLVX-Flag SW1116, PLVX-Flag-TOX SW1116, shCK HCT116, or shTOX-2 HCT116 cells (2 × 10^6^) were injected subcutaneously into 6-week-old nude male mice. Bodyweight (g) and tumor volume (mm^3^) were calculated weekly for each mouse in each group for 4 weeks. MC38-Flag and MC38-*Tox* cells were injected into the tail vein to establish a lung metastasis model in C57BL/6 mice. Three weeks later, mice were euthanized, and their lungs were collected and weighed. To test the effects of rapamycin and PD1 inhibitor, 0.2 million MC38-shCK and MC38-*shTox* cells were injected subcutaneously into 6-week-old male C57BL/6 mice. Animals were randomly divided into six groups (5 mice/group) after tumor cell injection to receive an intraperitoneal injection of PD1 inhibitor (USA, Bio-X cell, #BE0146, 12.5 mg/kg) or rapamycin (5 mg/kg) for 2 weeks. Rapamycin was injected every day for 2 weeks. PD1 inhibitor was injected twice a week for 2 weeks. Tumor volume (mm^3^) was calculated by the formula: volume = (width)^2^ × length/2.

### Statistical Analyses

Comparisons between groups were performed with SPSS version 20.0 (SPSS Inc., Chicago, IL, USA) using one-way analysis of variance, two-tailed Student's *t*-test, non-parametric tests, chi-squared test, or Fisher's exact test. The chi-square test (*n* ≥ 40 and T > 5), adjusted chi-square test (*n* ≥ 40 and 1 ≤ T < 5), or Fisher's exact test (*n* < 40 or T < 1) was used to determine the significance of difference between TOX and clinicopathological variables. Kaplan–Meier analysis with the log-rank test and the Cox proportional hazard model was used to determine the hazard ratio and 95% confidence interval for OS. All data are presented as mean ± standard deviation. Alpha (probability of making a type I error) for all statistical tests was 0.05. *P* < 0.05 was considered statistically significant.

## Discussion

**Figure 8:**
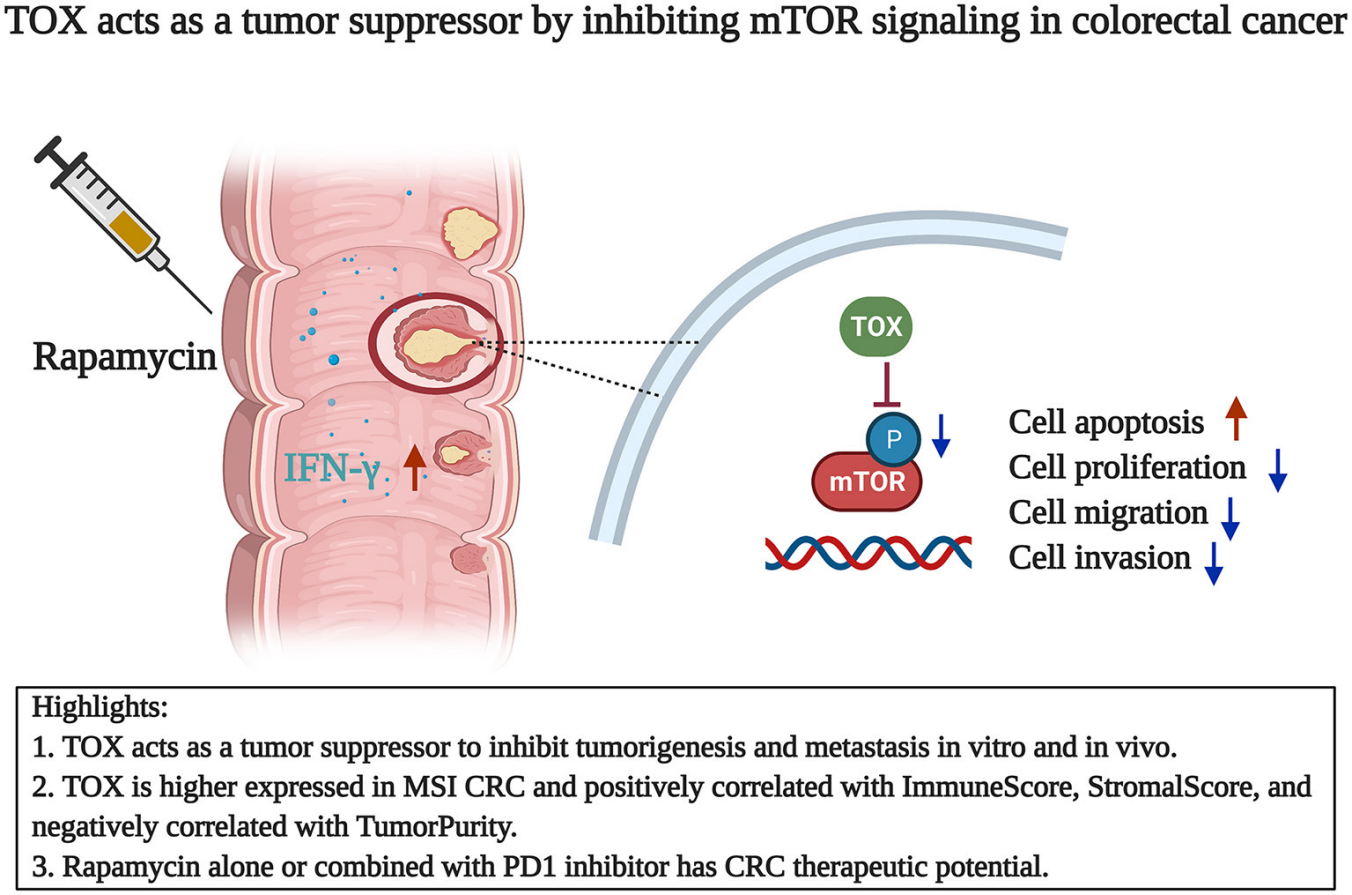
TOX acts as a tumor suppressor by inhibiting mTOR signaling in colorectal cancer. TOX acts as a CRC suppressor with decreasing expression and longer OS. TOX partly suppressed the mTOR signaling to inhibit cell proliferation, migration, invasion, and EMT process. Rapamycin alone or combined with PD1 inhibitor has therapeutic potential in CRC.

In this study, we showed that TOX appeared to act as a tumor suppressor in CRC: TOX expression was lower in CRC cells than para-CRC tissue. Tumor expression levels of TOX declined with increasing CRC tumor stage, and patients with low TOX had a poor prognosis. In contrast, patients with high TOX expression were mostly graded CMS3, indicating a good prognosis. Bioinformatics analysis showed that TOX expression negatively correlated with TumorPurity and positively correlated with MSI, ImmuneScore and StromalScore. Consistent with a role as a tumor-suppressor, TOX inhibited cancer cell proliferation and migration, likely by promoting the expression of epithelial markers and reducing mesenchymal markers, and promoted cancer cell apoptosis *in vitro*. Based on GSEA, loss of TOX results in activation of mTOR signaling in tumors. Inhibiting mTOR signaling *in vitro* partly reversed the aggressive cell proliferation and migration phenotype of TOX-deficient cells, indicating that TOX acts upstream of mTOR. The tumor-suppressive function of TOX on tumorigenesis was confirmed in mice using flank tumors to model tumorigenesis and tail vein injection to model metastasis. Additionally, rapamycin or PD1 inhibitor treatment suppressed the CRC growth of the *Tox*-deficient flank tumors. However, compared with rapamycin alone, the combined treatment cannot significantly reduce tumor volume (Figure 8).

Previous studies have shown that TOX is a critical regulator of tumor-specific T cell differentiation (26) and an initiator of the exhausted CD8^+^ T cell-specific epigenetic program (16). Downregulating TOX expression improves the anti-tumor function of CD8^+^ T cells, which can synergize with immune checkpoint suppression by anti-PD1, providing a promising strategy to enhance cancer immunotherapy (17). Alfei et al. found that TOX was a critical factor for the normal progression of T cell dysfunction and maintenance of exhausted T cells during chronic infection (27). Further, Huang et al. provided strong evidence that aberrant TOX activation is a critical oncogenic event for cutaneous T-cell lymphoma (28). Collectively, these results indicate that TOX plays a role similar to an oncogene in T cells.

However, despite the known roles of TOX in T cells, its regulatory role in CRC is largely unknown. MSI CRC has a higher TMB than MSS CRC (29, 30) and responds better to immunotherapy (31). The level and spatial distribution of CD3^+^ and CD8^+^ T cell infiltration differentiates four distinct solid tumor phenotypes: hot (or inflamed); altered, which can be excluded or immunosuppressed; and cold (or non-inflamed) (32). According to the definition of “hot” and “cold” tumors, the presence of exhausted CD8^+^ T cells in the TME may enable patients to respond to immunotherapy.

Our results indicate that TOX may play diverse and differential roles depending on the cell type and environmental context—despite of a previously established role for TOX to cause T cell exhaustion and inhibit tumor immunity, our comprehensive analysis of CRC indicates a novel tumor-suppressive role for TOX in CRC. We found that TOX appeared to act upstream, as an inhibitor, of the mTOR signaling pathway, which has known roles in cell proliferation, cell metabolism, and apoptosis (33, 34). Hyperactive mTOR signaling is a major cause of human diseases such as cancer (35), and the selective mTOR inhibitor rapamycin is an effective chemotherapy agent (36, 37). Our results also showed that rapamycin partly inhibited the cell proliferation and migration of shTOX HCT116 cells *in vitro*, indicating it may be particularly effective in targeting MSS tumors with low TOX expression. Our *in vivo* experiments provide further support for the tumor-suppressive role of TOX, where TOX overexpression inhibited tumor formation of SW1116 cells and lung metastasis of MC38 cells, and rapamycin significantly attenuated tumor progression of *shTox* MC38 cell-injected mice. Finally, we found that rapamycin or PD1 inhibitor had an anti-tumor therapeutic effect with more IFN-γ secretion in mice, suggesting potential clinical treatment benefit. However, the reports about the function of rapamycin on CD8^+^ T cells are controversial. Ruka et al. indicated that rapamycin inhibited the IFN-γ production by CD8^+^ T cells (38). Bak et al. showed that rapamycin treatment increased the frequency of antigen-specific CD8^+^ T cells and IFN-γ secretion (39). Our data suggested that rapamycin treat CRC by both suppress the tumor cells and enhance the function of infiltrated CD8^+^ cells.

This study is the first to show a tumor-suppressive role for TOX in CRC and that TOX is more highly expressed in MSI CRC patients than MSS CRC patients. TOX expression correlates with better survival of CRC patients and appears to inhibit CRC progression. Rapamycin or PD1 inhibitor suppresses tumorigenesis, likely in part by regulating signaling downstream of TOX. However, compared with rapamycin alone, the combined treatment cannot significantly reduce tumor volume compared to rapamycin alone. In conclusion, our *in vitro* and *in vivo* data indicate that TOX acts as a CRC tumor suppressor to inhibit tumorigenesis and metastasis, and rapamycin or combined with PD1 inhibition may be a promising treatment for CRC.

## Data Availability Statement

The original contributions presented in the study are included in the article/Supplementary Materials, further inquiries can be directed to the corresponding authors.

## Ethics Statement

The studies involving human participants were reviewed and approved by the Ethics Committee of Shanghai Jiao Tong University Affiliated Sixth People's Hospital. The patients/participants provided their written informed consent to participate in this study. The animal study was reviewed and approved by the Ethics Committee of Shanghai Jiao Tong University Affiliated Sixth People's Hospital.

## Author Contributions

MY contributed to the study methodology, performed experiments, statistical analysis, and writing. QH and CL performed the analysis and contributed to the project administration. ZJ, JS, and ZW collected the clinical samples and performed the investigation. DL validation of experimental details and research outputs. RL provided the study materials and instrumentation tools. HZ and BL conceived the overarching research goals and aims contributed to the supervision and acquisition of funding. All authors contributed to the article and approved the submitted version.

## Conflict of Interest

BL is a co-founder of Biotheus Inc and chairman of its scientific advisory board. The remaining authors declare that the research was conducted in the absence of any commercial or financial relationships that could be construed as a potential conflict of interest.
