# Supplementary figures and images for: TOX Acts as a Tumor Suppressor by Inhibiting mTOR Signaling in Colorectal Cancer
Source: Front Immunol. 2021 Apr 9;12:647540. doi: 10.3389/fimmu.2021.647540 (PMC8062716; doi:10.3389/fimmu.2021.647540)

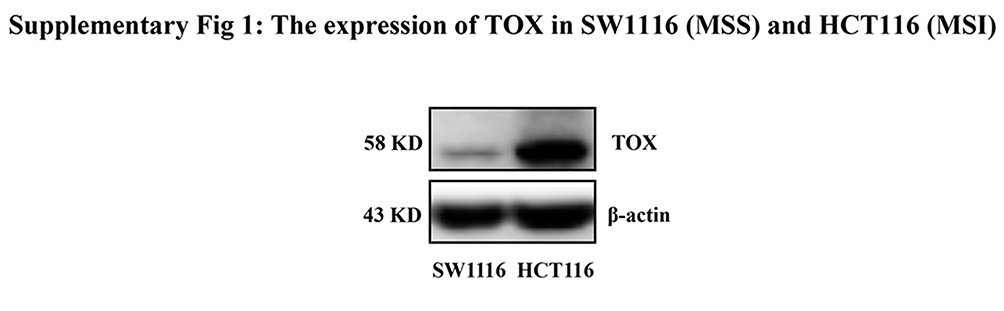

Supplement: Supplementary file 1 [file Image_1.TIF]

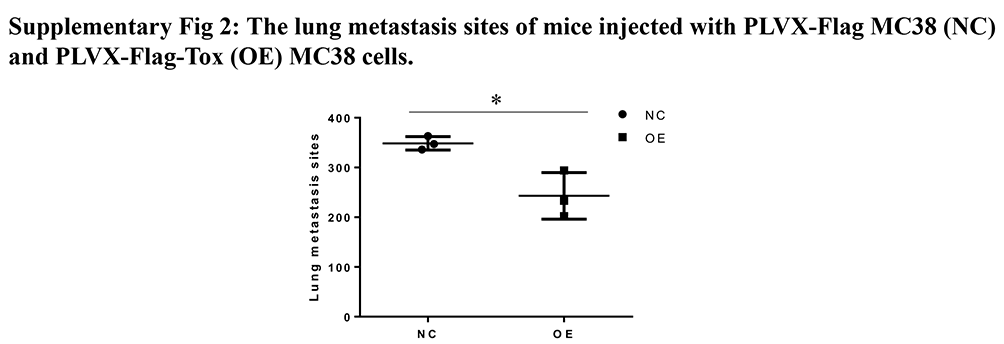

Supplement: Supplementary file 2 [file Image_2.TIF]
